# *Rickettsia parkeri* with a Genetically Disrupted Phage Integrase Gene Exhibits Attenuated Virulence and Induces Protective Immunity against Fatal Rickettsioses in Mice

**DOI:** 10.3390/pathogens10070819

**Published:** 2021-06-30

**Authors:** Esteban Arroyave, Ilirjana Hyseni, Nicole Burkhardt, Yong-Fang Kuo, Tian Wang, Ulrike Munderloh, Rong Fang

**Affiliations:** 1 Department of Pathology, University of Texas Medical Branch, Galveston, TX 77555, USA; esarroya@utmb.edu (E.A.); hyseni.ilirjana@gmail.com (I.H.); ti1wang@utmb.edu (T.W.); 2 Department of Entomology, University of Minnesota, St. Paul, MN 55108, USA; burkh032@umn.edu; 3 Department of Preventive Medicine and Community Health, University of Texas Medical Branch, Galveston, TX 77555, USA; yokuo@utmb.edu; 4 Department of Microbiology & Immunology, University of Texas Medical Branch, Galveston, TX 77555, USA; 5 Center for Biodefense and Emerging Infectious Diseases, University of Texas Medical Branch, Galveston, TX 77555, USA

**Keywords:** rickettsial mutant, attenuated virulence, protective immunity, fatal murine rickettsioses

## Abstract

Although rickettsiae can cause life-threatening infections in humans worldwide, no licensed vaccine is currently available. To evaluate the suitability of live-attenuated vaccine candidates against rickettsioses, we generated a *Rickettsia parkeri* mutant RPATATE_0245::pLoxHimar (named 3A2) by insertion of a modified pLoxHimar transposon into the gene encoding a phage integrase protein. For visualization and selection, *R. parkeri* 3A2 expressed mCherry fluorescence and resistance to spectinomycin. Compared to the parent wild type (WT) *R. parkeri*, the virulence of *R. parkeri* 3A2 was significantly attenuated as demonstrated by significantly smaller size of plaque, failure to grow in human macrophage-like cells, rapid elimination of *Rickettsia* and ameliorated histopathological changes in tissues in intravenously infected mice. A single dose intradermal (i.d.) immunization of *R. parkeri* 3A2 conferred complete protection against both fatal *R. parkeri* and *R. conorii* rickettsioses in mice, in association with a robust and durable rickettsiae-specific IgG antibody response. In summary, the disruption of RPATATE_0245 in *R. parkeri* resulted in a mutant with a significantly attenuated phenotype, potent immunogenicity and protective efficacy against two spotted fever group rickettsioses. Overall, this proof-of-concept study highlights the potential of *R. parkeri* mutants as a live-attenuated and multivalent vaccine platform in response to emergence of life-threatening spotted fever rickettsioses.

## 1. Introduction

In recent years, an increasing number of cases of rickettsial disease has been reported in several regions of North America where rickettsioses are endemic as well as in areas where rickettsioses have not commonly been diagnosed [1,2]. Currently, the estimated burden of disease is around 4000–6000 new cases annually, sharply up from 495 cases in 2000, including about 0.5% of cases with fatal outcomes from all spotted fever rickettsioses (https://www.cdc.gov/rmsf/stats/index.html) (21 June 2021). Life-threatening forms of spotted fevers (case fatality rate of 5–10% or more) are caused by *Rickettsia rickettsii* that is transmitted by wood ticks (*Dermacentor andersoni* and *Dermacentor variabilis*) in the Northern Rocky Mountains and South Central States, respectively, and brown dog ticks (*Rhipicephalus sanguineus*) in Arizona and neighboring regions of the USA [3]. Similarly, in South America, the incidence of Brazilian spotted fever has increased significantly, disproportionately affecting rural populations with poor access to health care services [4]. The Brazilian strain of *R. rickettsii* is transmitted by *Amblyomma* ticks [5], and the mortality rate is very high at 50% [6]. Due to the high case fatality rate, susceptibility of humans to infection via the aerosol route, and potential use as a bioweapon [7], an effective vaccine is urgently needed, but an FDA approved, and licensed vaccine is not available.

Historically, multiple strategies have been employed to develop a protective vaccine against rickettsioses, including the use of killed whole bacteria, subunits, recombinant antigens, and live attenuated strains [8,9,10,11]. The first live vaccine against *R. prowazekii* was generated after World War II by serial passage in hens’ eggs, yielding an attenuated strain, Madrid E, which prevented or reduced disease from louse-borne typhus, although it caused mild typhus in about 14% of vaccinated persons [10,12,13]. Subunit antigens have also been developed but not licensed due to the concerns on either protective efficacy or safety [14,15,16]. The failure to develop a vaccine against rickettsioses is mainly attributed to inadequate knowledge of the protective antigens, virulence mechanisms of pathogenic rickettsial species, and incomplete understanding of vaccine-induced immunity [17,18].

Analysis of rickettsial gene function in heterologous systems through standard cell-molecular analyses, genome sequencing and bioinformatic comparisons has facilitated elucidation of the role of genetic components [19,20,21,22,23,24,25]. Although creation of a defective phenotype induced through mutagenesis and rescue through complementation would provide definitive assignment of function, this has rarely been achieved for rickettsiae [18,26]. Direct genetic manipulation and the refinement of genetic manipulation methods for all obligately intracellular bacteria, including rickettsiae, have proven exceedingly difficult [27], mostly because these microorganisms only proliferate inside of host cells, and thus only invasion- and replication-competent bacterial mutants can be obtained. In addition, phenotypic analysis of rickettsial mutants, particularly *in vivo*, has been only partially successful. For example, a knock-out mutation of *ompA* in *R. rickettsii*, a gene long considered to encode a major virulence factor, did not result in a phenotypic change [17]. In addition, non-virulent *R. rickettsii* transformed with a shuttle vector expressing the RARP-2 gene encoding an ankyrin-repeat protein from virulent *R. rickettsii* displayed a lytic plaque phenotype while it is not virulent to guinea pigs [18,25].

*R. parkeri* is a spotted fever agent transmitted by Gulf coast ticks in the South-Central US and Gulf coast states [28], which causes a relatively mild and infrequently diagnosed infection referred to as American boutonneuse fever. Based on the few diagnosed cases of *R. parkeri* rickettsioses to date [29], *R. parkeri* could be considered naturally attenuated; however, its virulence to animals or potential to elicit severe illness in immune-compromised persons is unknown [30]. We have generated a set of *R. parkeri* mutants (strain Tate’s Hell, accession Nr.:LAOO01000001) using random transposon mutagenesis to insert genes encoding fluorescent and selectable markers. Analysis of this library will identify rickettsial genes required for infection of mammals and pathogenicity, and guide identification of desirable mutants, as well as provide a valuable resource to the community of rickettsiologists. Among these mutants, we selected a *R. parkeri* mutant with a modified Himar1 transposon insertion in a phage-integrase gene that displayed an attenuated phenotype. *R. parkeri* mutant RPATATE_0245::pLoxHimar, designated “3A2”, was evaluated as a vaccine candidate against spotted fever rickettsioses using cellular and animal models.

The present study demonstrated the immunogenicity and efficacy of *R. parkeri* 3A2 by the potent *Rickettsia*-specific antibody response and Th1 effector cytokine in sera, and the complete survival of immunized mice upon subsequent challenge with WT *R. parkeri* or *R. conorii*. Our findings in these proof-of-concept studies highlighted the potential for the development of an effective single dose live-attenuated vaccine against more than one life-threatening rickettsiosis. Our data also provided insight into vaccine-induced immunity against severe or fatal rickettsial diseases.

## 2. Results

### 2.1. Mapping of Himar1 Transposon Insertion Site and Phenotypic Characterization of R. parkeri 3A2

**Figure 1:**
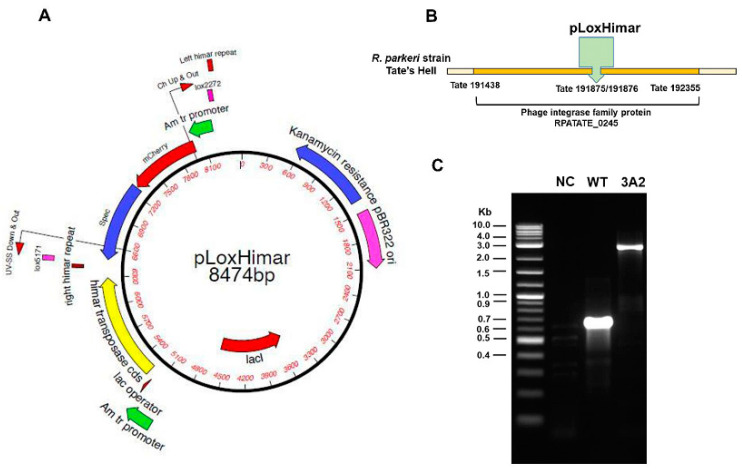
Engineering of obligately intracellular bacteria *R. parkeri* by random mutagenesis. (**A**) The Himar1 plasmid was adapted to include the mCherry (red fluorescent marker) and *aadA* genes flanked by mismatched lox5171 and lox2272 sites (designated “lox”) to facilitate excision of the transposon. *R. parkeri*, strain Tate’s Hell, was electroporated with the resulting pCis himar cherry A7 lox plasmid (pLoxHimar). (**B**) The insertion site of the pLoxHimar transposon in the genome of *R. parkeri* mutant 3A2 was identified by sequencing. (**C**) Confirmation of the insertion site by PCR. Primers were designed to target genomic sequences flanking the transposon. DNA from uninfected Vero cells (negative control, NC) and parent WT *R. parkeri*-infected Vero cells served as controls. The 2-Log DNA Ladder (0.1–10.0 kb) (New England BioLabs) was included to estimate the size of the amplicons.

Although the Himar1 transposase system has been shown to be an effective approach for random mutagenesis of Rickettsiales [31,32], its efficiency for transformation of *Rickettsia* spp. is still relatively low when compared to non-obligately intracellular organisms [33]. We generated a *R. parkeri* RPATATE_0245::pLoxHimar mutant, designated “3A2”, by electroporation of *R. parkeri* strain Tate’s Hell with the pCis himar cherry A7 lox (pLoxHimar) plasmid (Figure 1A). The specific insertion locus for *R. parkeri* 3A2 was assigned by insertion site cloning of BglII fragments into pGEM and selection of positive clones with spectinomycin/streptomycin. Sequencing of positive clones showed that the pLoxHimar transposon was inserted at nucleotide position 191875/191876 in the genome of *R. parkeri* strain Tate’s Hell. The mutation interrupted the open reading frame (Tate 191438–Tate 192355) of a gene encoding a phage integrase family protein (RPATATE_0245). A pair of primers was designed based on the genome sequence and used to amplify both WT and *R. parkeri* 3A2 (Figure 1B). As shown in Figure 1C, the sizes of amplified products of *R. parkeri* 3A2 and *R. parkeri* WT were 2506 and 612 bp, respectively. The greater size of the amplicon in *R. parkeri* mutant 3A2 compared to WT *R. parkeri* indicated that the transposon was inserted within the gene RPATATE_0245.

**Figure 2:**
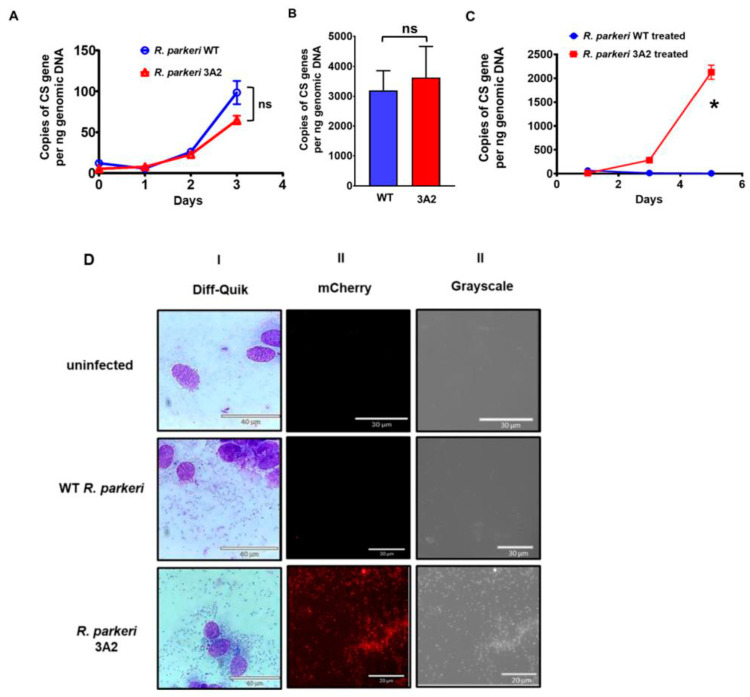
Antibiotic resistance and mCherry expression in *R. parkeri* RPATATE_0245::pLoxHimar (3A2). Vero cells were infected with *R. parkeri* WT and 3A2 at the same multiplicity of infection (MOI) of 2. Infected cells were cultured with either plain medium (**A**,**B**), or with medium containing 100 µg/mL spectinomycin (**C**). At the indicated time points (**A**,**C**) or day 5 post infection (p.i.) (**B**), cells were collected for quantifying the kinetic intracellular growth of these rickettsiae by quantitative real-time PCR amplifying citrate synthase (CS) gene. (**D**) Vero cells were infected with *R. parkeri* WT and 3A2. Cells were collected when the infection rate reached greater than 80%, as shown by the light microscopy after Diff-Quik staining (I). A drop of cell suspension under a cover slip was imaged under mCherry fluorescence (II) using an Echo Revolve Microscope (Echo Inc., San Diego, CA, USA). In addition, *R. parkeri* WT and 3A2 were also visualized under grey scale (III). Uninfected Vero cells served as negative controls. Scale bars were shown as labeled. ns, not statistically significant. *, *p* < 0.01.

Because the transposon included the *aadA* gene for selection, we tested whether *R. parkeri* 3A2 expressed antibiotic resistance (Figure 2A–C). First, in the regular growth medium, *R. parkeri* 3A2 replicated at a rate similar to *R. parkeri* WT over three days (Figure 2A) (*p* = 0.5366 by Friedman test). No significant difference in the concentrations of *R. parkeri* WT vs 3A2 in Vero cells on day 5 p.i. in the absence of spectinomycin (Figure 2B) (*p* = 0.8248 by Mann-Whitney test). The growth kinetics of *R. parkeri* 3A2 were comparable to WT *R. parkeri* in Vero cells, suggesting that this mutant does not have any deficiency in replication. In the presence of spectinomycin, *R. parkeri* WT did not show any significant growth while numbers of *R. parkeri* 3A2 progressively increased throughout the assay period of five days with significant difference from those of *R. parkeri* WT (Figure 2C) (*p* = 0.0007 by Friedman test). Thus, these results confirmed the antibiotic resistance phenotype of *R. parkeri* 3A2. Furthermore, *R. parkeri* 3A2 expressed mCherry and fluoresced red when viewed under a fluorescence microscope with a Texas Red filter, while *R. parkeri* WT did not show any fluorescence, although these two strains were at a very similar infection level as shown by Diff-Quik staining (Figure 2D).

### 2.2. Genetic Disruption of Phage Integrase Family Protein RPATATE_0245 of R. parkeri Results in Virulence Attenuation In Vitro

**Figure 3:**
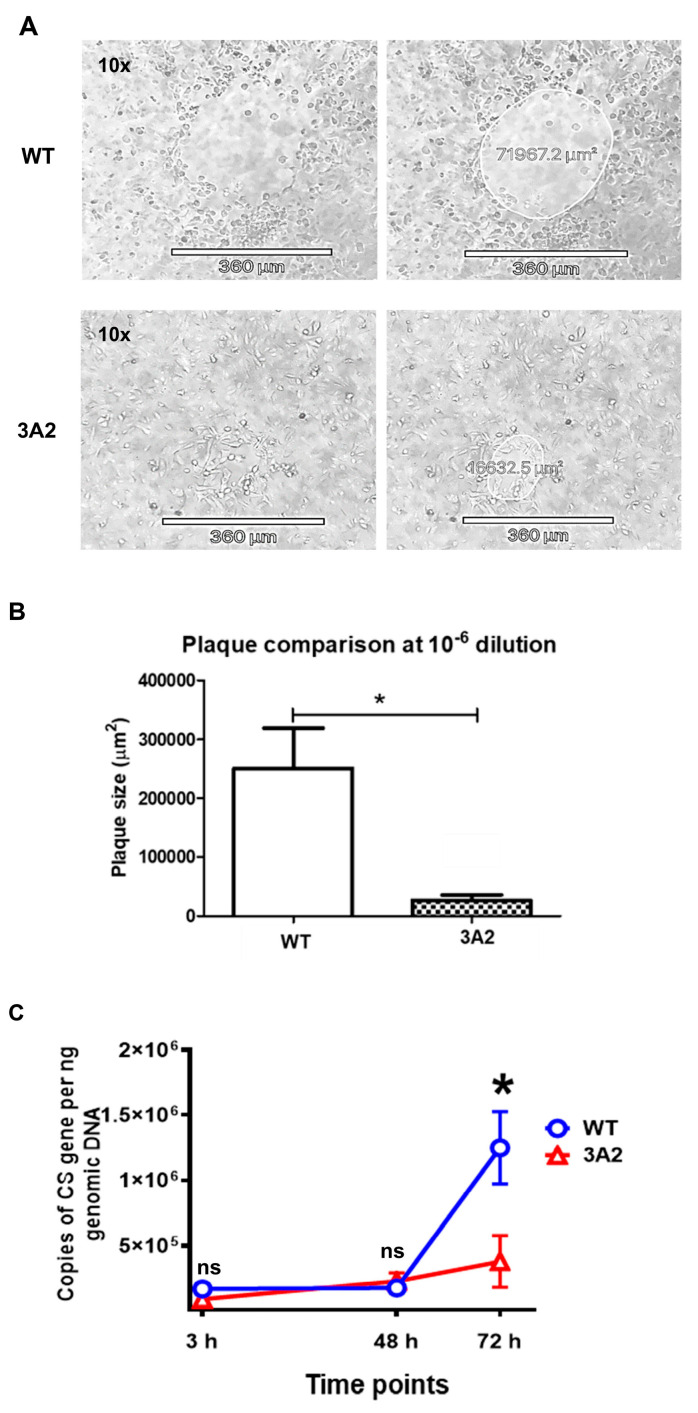
Evaluation of the virulence of *R. parkeri* 3A2 *in vitro*. Plaque assay was performed by the procedures described in Materials and Methods. The plaques generated by *R. parkeri* WT and 3A2 were monitored, imaged and measured. During days 3–5 p.i., the representative plaques generated by *R. parkeri* WT and 3A2 at the same dilution on the same day of infection are shown in (**A**). On the next day, plaques were determined and analyzed (**B**). (**C**) Human THP-1 cells were differentiated to macrophages using phorbol myristate acetate (PMA), and infected with *R. parkeri* WT and 3A2 at an MOI of 5. At 3 h, 48 h and 72 h p.i., the intracellular concentrations of *R. parkeri* WT and 3A2 were determined by quantitative real-time PCR amplifying the citrate synthase (CS) gene. Data represent two independent experiments with similar results. ns, not statistically significant. *, *p* < 0.05.

To determine the effect of the interruption of RPATATE_0245 on *R. parkeri* virulence *in vitro*, we compared the size of plaques generated by WT *R. parkeri* and *R. parkeri* 3A2. Despite the similar growth rates, *R. parkeri* 3A2 exhibited a small-plaque phenotype in Vero cells compared to WT *R. parkeri* (Figure 3A). The plaque sizes generated by *R. parkeri* 3A2 in Vero cells on day 5 p.i. were significantly smaller at a 10^ 6 dilution than those of WT *R. parkeri* at the same dilution (Figure 3B). Previous studies have shown that virulent *R. conorii* differs in growth kinetics in phagocytes from avirulent *R. montanensis* [34]. We infected human macrophage-like cells with the same amounts of WT *R. parkeri* and mutant 3A2. Compared to WT *R. parkeri*, the concentrations of *R. parkeri* 3A2 in these professional phagocytes were significantly less at 72 h p.i., but not at the earlier time points (Figure 3C).

### 2.3. Evaluation of Safety and Immunogenicity of R. parkeri 3A2 in a Mouse Model of Rickettsioses

The use of *R. parkeri* Atlantic Rainforest strain to infect C3H/HeN mice provides an excellent model to study the pathogenesis of rickettsioses at the biosafety level 2 [35]. We first determined whether *R. parkeri* strain Tate’s Hell can establish a dose-dependent mouse model for spotted fever rickettsioses. Indeed, C3H/HeN mice inoculated intravenously (i.v.) with a low dose (2 × 10^7 or 4 × 10^7 copies of CS gene per mouse) of WT *R. parkeri* showed weight loss and illness, including ruffled fur, lethargy, and decreased movement, and survived (Supplementary Figure S1A,B). In response to high doses ranging from 1 × 10^8 to 4 × 10^8 copies of CS gene per mouse of WT *R. parkeri*, mice developed more severe illness including dramatic weight loss, hunched posture, or even became moribund (Supplementary Figure S1A,B). The severity of illness in mice correlated with the inoculum and rickettsial load in tissues such as liver and the dose of inoculum (Supplementary Figure S1C). Thus, we demonstrated that C3H/HeN mice infected with WT *R. parkeri* strain Tate’s Hell provides a dose-dependent mouse model of spotted fever rickettsioses.

**Figure 4:**
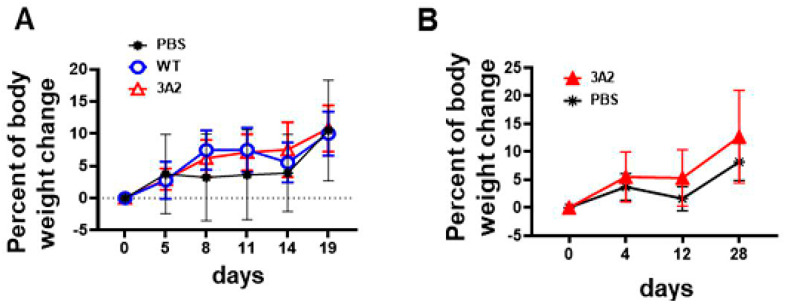

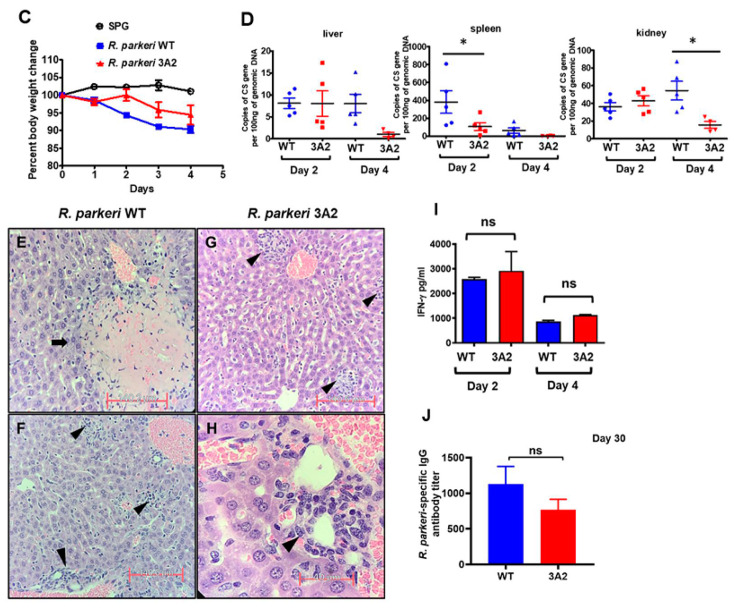
Evaluation of the virulence and immunogenicity of *R. parkeri* 3A2 in mice. C3H/HeN mice were inoculated i.d. (immunized) with *R. parkeri* 3A2 at a dose of 1 × 10^3 (**A**) or 1 × 10^5 (**B**) copies of CS gene per mouse. Mice inoculated with PBS and the same dose of *R. parkeri* WT served as negative and positive controls, respectively. Mice were monitored for skin lesions at the injection site and weight loss. (**C**) C3H/HeN mice were inoculated i.v. with *R. parkeri* 3A2 at a dose of 3 × 10^5 copies of CS genes per mouse. Mice inoculated i.v. with sucrose-phosphate-glutamate (SPG) buffer and the same dose of *R. parkeri* WT served as negative and positive controls, respectively. After infection, mice were monitored, and weight loss was calculated. On days 2 and 4 p.i., mice were euthanized. Tissues were collected to determine the loads of rickettsiae by quantitative real-time PCR amplifying citrate synthase (CS) gene (**D**). On day 4 p.i., livers were collected and processed for histopathological analysis (**E–H**). Coagulative necrosis lesions (arrows) were present only in livers of mice infected with WT *R. parkeri* (**E**), but not *R. parkeri* 3A2. Foci of inflammatory cells, apoptotic cells, and perivascular inflammation (arrowheads) were observed in livers of all infected mouse groups (**F–H**). In (**E–G**), scale bar = 140 µm. In H, scale bar = 40 µm. On day 4 p.i., sera were collected from mice infected i.v. with *R. parkeri* WT or 3A2. The concentrations of IFN-γ in serum were determined by ELISA (**I**). On day 30 post immunization, serum was collected. The titer of *R. parkeri* WT-specific IgG antibody was determined by indirect immunofluorescent assay (IFA) (**J**). Each group included 4–10 mice. Data represent two independent experiments with similar results. * *p* < 0.05; ns, not statistically significant.

Next, to evaluate the virulence or safety of *R. parkeri* mutants *in vivo* as potential vaccine candidates, we immunized C3H/HeN mice with a single dose of *R. parkeri* 3A2 i.d.. Compared to mice immunized with phosphate-buffered saline (PBS) or the same dose of WT *R. parkeri*, a single dose of immunization of *R. parkeri* 3A2, at either a low or a high dose, did not cause any systemic sickness, skin lesion at the injection site, or weight loss (Figure 4A,B). To further study the *in vivo* virulence of *R. parkeri* mutant, we inoculated C3H/HeN mice with *R. parkeri* 3A2 i.v.. Mice i.v. inoculated with *R. parkeri* 3A2 showed weight loss compared to mock-infected mice (Figure 4C). The concentrations of *R. parkeri* 3A2 in tissues were significantly less in spleen on day 2 p.i. and in kidney on day 4 p.i., compared to mice infected with WT *R. parkeri* at the same dose (Figure 4D). *R. parkeri* 3A2 in tissues were nearly non-detectable on day 4 p.i. in tissues including liver, spleen, and kidney. Histopathological analysis of liver in mice infected with WT *R. parkeri* showed coagulative necrosis of hepatic parenchyma, which was not found in mice infected with *R. parkeri* 3A2 (Figure 4E–H). However, perivascular inflammation and foci of inflammatory cells were observed in livers of mice infected with *R. parkeri* 3A2 (Figure 4G,H). These results suggest that the *in vivo* virulence of *R. parkeri* 3A2 is attenuated compared to WT *R. parkeri* when it was inoculated i.v., although it also caused non-extensive pathological changes in tissues, which most likely results from the host immune response controlling rickettsiae.

To evaluate the immunogenicity of this mutant, we infected C3H/HeN mice with *R. parkeri* 3A2 i.v.. On both days 2 and 4 p.i., the levels of IFN-γ in sera of mice infected with *R. parkeri* 3A2 were comparable to those in mice infected with WT *R. parkeri* (Figure 4I). On day 30 p.i., the titer of *R. parkeri*-specific IgG antibody in serum of mice i.v. infected with *R. parkeri* 3A2 was not significantly different compared to mice infected with *R. parkeri* WT (Figure 4J), with a titer of approximately 1:1000. These results suggest that *R. parkeri* 3A2 induces potent type 1 cellular immunity and B cell response *in vivo*, most likely triggered by the effective immunogenicity of these bacteria.

### 2.4. A Single Dose Immunization of R. parkeri 3A2 Induces Potent and Non-Transient Antibody Response Associated with Rickettsial Clearance In Vivo

**Figure 5:**
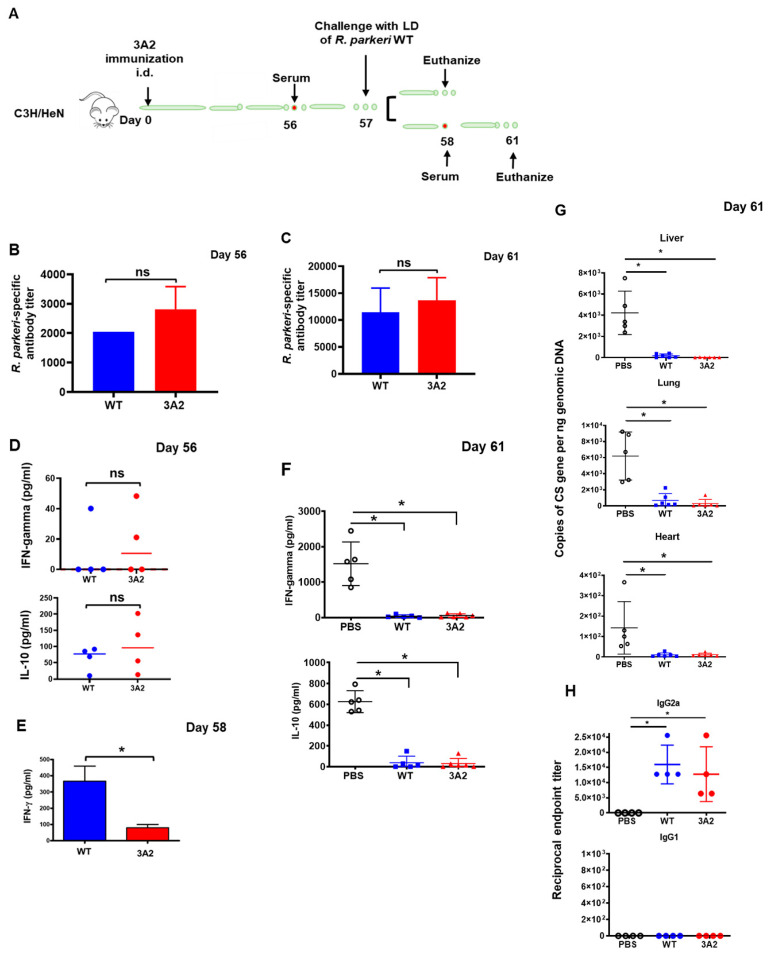
A single dose immunization of *R. parkeri* 3A2 stimulated a potent antibody response against WT *R. parkeri* in mice, accompanied by undetectable rickettsial load *in vivo* upon challenge. C3H/HeN mice were immunized i.d. with live *R. parkeri* 3A2 at a single dose of 10^3 copies of CS gene per mouse. Mice immunized i.d. with PBS or with the same dose of *R. parkeri* WT served as negative and positive controls, respectively. Sera were collected at different time points, as indicated in (**A**). On day 57 post immunization, mice were infected i.v. with a lethal dose (LD) (1 × 10^8 copies of CS gene per mouse) of *R. parkeri* WT. Mice were euthanized on day 58 or 61 post immunization. The antibody response stimulated by *R. parkeri* 3A2 was determined on days 56 (**B**) and 61 (**C**) post immunization by the titer of *R. parkeri*-specific IgG. The levels of IFN-γ and/or IL-10 in serum of these immunized mice on days 56 (**D**), 58 (**E**) and 61 (**F**) post immunization were determined by ELISA. (**G**) On day 61 post immunization (4 days p.c.), the loads of rickettsiae in mouse tissues, including lung, liver, and heart, were determined by quantitative real-time PCR amplifying the citrate synthase (CS) gene. (**H**) Serum was collected on day 61 post immunization. The IgG isotype was determined by ELISA as described in the materials and methods. Each group included 6–10 mice. ns, not statistically significant; *, *p* < 0.05.

To further evaluate the potential of *R. parkeri* 3A2 as a vaccine candidate against rickettsial diseases, we immunized C3H/HeN mice with *R. parkeri* 3A2 i.d. followed by assessment of the host immune response upon i.v. challenge 8 weeks later (Figure 5A). As controls, mock-immunized mice did not produce IgG antibody against *R. parkeri* (Supplementary Figure S2). On day 56 post immunization, with a single dose of *R. parkeri* 3A2, the level of *R. parkeri*-specific antibodies was comparable to that induced by WT *R. parkeri* (Figure 5B), at a titer of approximately 1:3000. Four days after challenge of mice with a lethal dose of WT *R. parkeri*, the titer of *R. parkeri*-specific antibody response increased to 1:15,000 in mice previously immunized with either WT *R. parkeri* or *R. parkeri* 3A2 (Figure 5C). 

As expected, we did not detect a significant level of IFN-γ in sera of mice immunized with either *R. parkeri* WT or 3A2 one day before challenge with a lethal dose of *R. parkeri* i.v. (Figure 5D). One day after lethal challenge, greater production levels of IFN-γ in sera were detected in mice immunized with WT *R. parkeri* compared with *R. parkeri* 3A2 (Figure 5E), which is either associated with the *in vivo* rickettsial loads in tissues or the levels of type 1 immune response. Four days after lethal challenge, we found significant levels of IFN-γ and IL-10 in sera of mock-immunized mice, but not in mice immunized with either WT *R. parkeri* or *R. parkeri* 3A2 (Figure 5F). Strikingly, we did not detect a significant amount of rickettsiae in tissues, including liver, lung and heart, of mice immunized with WT *R. parkeri* or *R. parkeri* 3A2, on day 4 post challenge (p.c.) (Figure 5G). As controls, a significant amount of rickettsiae was detected in tissues of mock-immunized mice (Figure 5G), suggesting that a single dose of immunization of *R. parkeri* 3A2 induces a potent host immune response resulting in the effective clearance of rickettsiae in less than four days.

In mice, Th1-dependent IFN-γ induces the production of IgG2a, whereas the Th2 cytokine IL-4 stimulates the expression of IgG1, rendering each isotype an indicator of the underlying Th cell response [36]. To further evaluate the immune response initiated by immunization with *R. parkeri* 3A2, we determined the isotype of IgG antibodies. Mice immunized with a single dose of *R. parkeri* 3A2 showed a greater endpoint titer of IgG2a (mean = 10,000), compared to mock-immunized mice, and was not significantly different from mice immunized with WT *R. parkeri* (Figure 5H). Interestingly, no significant IgG1 antibody response was detected (Figure 5H). These results suggest that *R. parkeri* 3A2 immunization leads to an antibody response associated with robust Th1 immunity against rickettsiae.

### 2.5. A Single Dose Immunization of R. parkeri 3A2 Confers Protection against Fatal Murine Spotted Fever Rickettsioses

**Figure 6:**
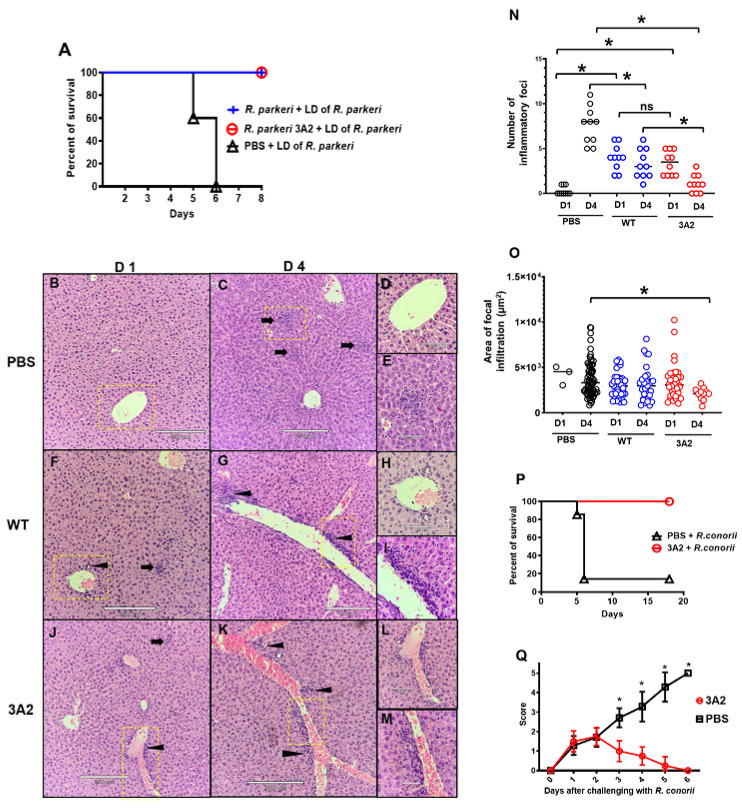
A single dose immunization of *R. parkeri* 3A2 conferred complete protection against fatal murine spotted fever rickettsioses. C3H/HeN mice were immunized i.d. with live *R. parkeri* 3A2 at a single dose of 10^3 copies of CS gene per mouse. Mice mock-immunized i.d. with PBS or *R. parkeri* WT at the same dose as *R. parkeri* 3A2 served as negative and positive controls, respectively. On day 57 post immunization, mice were challenged i.v. with a lethal dose (LD) (1 × 10^8 copies of CS gene per mouse) of WT *R. parkeri*. (**A**) After challenge, host survival was monitored daily. (**B**–**M**) On days 1 (D1) and 4 (D4) p.c., mice were euthanized. Liver was collected and processed for histopathological analysis. Figures shown represent images captured at x10 (scale bar = 250 µm) and ×20 (scale bar = 100 µm) (**B**–**M**), respectively. Arrows represent focal cellular infiltration in hepatic lobules. Arrowheads represent perivascular inflammation. To quantify the histopathological changes, the inflammatory foci in livers of each group of mice were visualized at ×10 and counted in at least 10 randomly selected fields (**N**). The area of the selected inflammatory foci was measured and analyzed using an Echo Revolve Microscope (**O**). Each circle represented one inflammatory focus. In mice immunized with PBS, no or minimum inflammatory infiltration was observed on day 1 p.c., and inflammatory foci were found on day 4 p.c. without accompanying perivascular inflammation (**B**–**E**). Compared to mice immunized with *R. parkeri* WT (**F**–**I**), mice immunized with *R. parkeri* 3A2 (**J**–**M**) showed fewer inflammatory foci in liver on day 4 p.c. (**N**,**O**). Another group of naïve C3H/HeN mice were immunized i.d. with *R. parkeri* 3A2 at a single dose of 10^5 copies of CS gene per mouse (**P**,**Q**). Mice immunized i.d. with PBS served as negative controls. On day 54 post immunization, mice were infected i.v. with *R. conorii* at a dose of 4.6 × 10^4 PFU per mouse. Host survival (**P**) and illness (**Q**) were monitored daily. Illness scores were calculated according to Table 1. Each group included 6–10 mice. ns, not significantly different by statistical analysis; *, *p* < 0.05.

Finally, we evaluated the efficacy of *R. parkeri* 3A2 in providing host protection from lethal challenge of *R. parkeri* or a high dose challenge with *R. conorii*, which are the dose-dependent mouse models of spotted fever rickettsioses [37]. On day 57 post immunization with a single dose of *R. parkeri* 3A2 (Figure 5A), mice were challenged with a lethal dose of WT *R. parkeri* i.v. Lethal challenge caused 100% death in mock-immunized mice (Figure 6A). Strikingly, 100% of mice immunized with *R. parkeri* 3A2 survived upon lethal challenge with *R. parkeri* (Figure 6A), as did mice immunized with *R. parkeri* WT.

Next, we evaluated the histopathological changes in mice immunized with *R. parkeri* 3A2 followed by lethal challenge with *R. parkeri* WT to further assess the safety of this mutant as a vaccine candidate (Figure 6B–O). On day 1 p.c., mice immunized with PBS showed no or minimal inflammatory infiltration in liver (Figure 6B,D,N,O). Mice immunized with either *R. parkeri* 3A2 or *R. parkeri* WT showed focal inflammatory cellular infiltration and perivascular inflammation in liver on day 1 p.c. (Figure 6F,H,J,L). The number of inflammatory foci and area of the inflammatory infiltrates in liver of mice immunized with WT *R. parkeri* were not significantly different compared to *R. parkeri* 3A2 on day 1 p.c. (Figure 6N,O).

On day 4 p.c., mice immunized with PBS showed inflammatory foci in liver with significantly greater frequency and comparable area of focal cellular infiltration compared to mice immunized with *R. parkeri* WT (Figure 6C,E,N,O). In contrast, compared to *R. parkeri* WT (Figure 6G,I,N,O), mice immunized with *R. parkeri* 3A2 showed significantly reduced frequency of inflammatory foci in liver (Figure 6K,M–O). Compared to mock-immunized mice, both the frequency of inflammatory foci and area of focal infiltration were significantly reduced in mice immunized with *R. parkeri* 3A2 (Figure 6N,O). These results suggest that immunization with *R. parkeri* 3A2 confers 100% protection against lethal challenge with WT *R. parkeri* accompanied by ameliorated pathological changes.

*R. conorii*-infected C3H/HeN mice have been used as an animal model for Rocky Mountain spotted fever, which is the life-threatening rickettsial infection in the US, due to the consistence in representative pathology [37]. In order to determine whether *R. parkeri* 3A2 potentially confers protection against Rocky Mountain spotted fever, we challenged mice immunized with *R. parkeri* 3A2 with a high dose of *R. conorii* i.v. Approximately 90% of mock-immunized mice succumbed to infection with *R. conorii* while 100% of mice immunized with *R. parkeri* 3A2 survived (Figure 6P). Upon challenge with *R. conorii*, mock-immunized mice showed severe sickness including ruffled fur, hunched back, squinty eyes and became moribund (Figure 6Q). In contrast, mice immunized with *R. parkeri* 3A2 showed minimal sickness within two days after challenge, and rapidly recovered with normal activity on day 3 p.c. (Figure 6Q). These results provided the proof-of-concept evidence that a *R. parkeri* mutant, with significantly attenuated phenotype, can confer significant protection against one of the most severe rickettsial diseases.

## 3. Discussion

Tick-borne diseases (TBDs) have become a global public health challenge and will affect over 30% of the global population by 2050 [38]. Vaccines are the most effective means to prevent infectious diseases. Despite many years of attempts, a licensed vaccine against tick-borne rickettsial diseases is still not available. We developed *R. parkeri* 3A2 by engineering the genome of *R. parkeri* with an insertion of a modified transposon in the open reading frame of a phage integrase protein, which significantly attenuated the virulence of WT *R. parkeri*. As a proof-of-concept, we demonstrated that a single-dose of live-attenuated rickettsial mutant confers protection against more than one disease caused by tick-borne rickettsial pathogens. A single vaccination in mice induced a potent and durable humoral response, and type I recall immune response, which are associated with complete protection against the subsequent challenge with two important human pathogens, *R. parkeri* and *R. conorii*.

The virulence of *R. parkeri* 3A2 is highly attenuated, as demonstrated by rapid clearance of rickettsiae from the tissues of infected mice, inability to replicate in human macrophage-like cells, transient and mild sickness as well as significantly ameliorated pathological changes in mice. Safety is a principal issue for all live-attenuated vaccines. Previous studies have shown that a single nucleotide insertion in the methyltransferase gene changes highly virulent *R. prowazekii*, the etiologic agent of epidemic typhus, into avirulent *R. prowazekii* Madrid E strain [39,40,41]. Although *R. prowazekii* E strain shows low virulence for humans and experimental animals [42], it is not used as a vaccine due to its reversion to virulence. Unlike *R. prowazekii* E strain, which was fortuitously obtained during laboratory passage, *R. parkeri* 3A2 was generated using specific mutagenesis and antibiotic selection. Furthermore, WT *R. parkeri* is a rickettsial species of low virulence that causes mild illness in humans. Compared to low virulence WT *R. parkeri*, *R. parkeri* 3A2 caused markedly reduced size of plaques *in vitro* (Figure 3) and significantly ameliorated sickness accompanied by rapid bacterial clearance as well as causing fewer lesions in tissues of infected mice (Figure 4). Importantly, mice immunized with *R. parkeri* 3A2 conferred 100% protection against subsequent lethal challenge with WT *R. parkeri* associated with elimination of rickettsiae within four days, whilst retaining a minimum level of pathological changes in tissues (Figure 6). We anticipate that this mutant is stable, but we are serially passing this mutant in cell culture with periodic examination of its genotype to verify this assumption.

Although markedly attenuated, *R. parkeri* 3A2 stimulated a robust IgG antibody and IFN-γ recall response in mice following lethal challenge with rickettsiae. Epidemiological studies have never reported any repetitive infection with rickettsiae in humans. This is indirect evidence that people who survive rickettsial infection may develop long-lasting protective memory immunity. Based on these observations, we employed mice immunized with WT *R. parkeri* as a control for optimal vaccine-induced immunity against tick-borne rickettsial diseases. The IgG antibody responses in mice infected or immunized with *R. parkeri* 3A2 were as robust and durable as those in mice infected or immunized with the same dose of WT *R. parkeri* (Figure 4J and Figure 5B,C). These results suggest that the immunogenicity of *R. parkeri* 3A2 is potent enough to elicit strong and non-transient humoral immunity, even via a single dose of vaccination. However, the titer of rickettsiae-specific IgG antibody was significantly increased after lethal challenge with rickettsiae, suggesting that further optimizing immunization dose and/or frequency may lead to a saturated antibody titer conferring ideal protection against rickettsioses. Remarkably, the isotype of IgG antibody stimulated by immunization with *R. parkeri* 3A2 was exclusively IgG2a, without IgG1, suggesting predominant Th1 cellular immunity (Figure 5H). Indeed, the significant IFN-γ level was detected in sera of mice immunized with *R. parkeri* 3A2 one day after the lethal challenge (Figure 5E), but not before challenge (Figure 5D), suggesting a Th1 memory immune response. We did not detect significant amount of rickettsiae in tissues of vaccinated mice on day 4 p.c. (Figure 5G), which may result from limited detection of rickettsiae *in vivo* by quantitative real-time PCR or rapid clearance of *R. parkeri* by the robust Th1 recall response and potent antibody response. The significantly lower levels of IFN-γ in serum of mice immunized with *R. parkeri* 3A2 compared to WT (Figure 5E) could be due to the lower rickettsial load in tissue or reduced immunogenicity of *R. parkeri* 3A2 in stimulating Th1 memory immunity. Further studies are required to reveal whether the lack of detection of IFN-γ in serum of mice immunized with *R. parkeri* WT or 3A2 on day 4 p.c. (Figure 5F) results from a minimum rickettsial load in animal tissues. Nevertheless, *R. parkeri* 3A2 conferred complete protection against lethal challenge in mice (Figure 6), which is associated with, at least, the potent IgG antibody response.

Genetic disruption of a gene encoding a phage integrase in *R. parkeri* markedly attenuated its virulence phenotypically. Phages are viruses that infect bacteria [43]. Phage integrases are enzymes mediating efficient site-specific recombination between two different sequences specific on a genomic scale [43]. Phage integrases are some of the few tools for the precise manipulation of the genome in both eukaryotic and prokaryotic cells [44]. It remains unknown why a phage integrase is important for the virulence of a rickettsial species. Among rickettsial pathogens with variable virulence, evaluation of its virulence has been performed either by clinical and epidemiological studies, or in experimental animals. These approaches are time-consuming and labor-intensive. In our studies, *R. parkeri* 3A2 showed reduced virulence *in vitro* using the cellular models (Figure 3). Although future studies are required to validate these approaches for screening the virulence of *Rickettsia*, our *in vitro* results on *R. parkeri* 3A2 correlates well with the virulence profile in mice (Figure 4). Thus, our studies highlighted the convenience and feasibility of an *in vitro* approach for assessment of virulence of an emerging-rickettsial species in an experimental setting.

In summary, using *R. parkeri* Tate’s Hell WT as a parent strain, we developed an innovative live attenuated rickettsial mutant to serve as a potential vaccine candidate against more than one spotted fever rickettsiosis. *R. parkeri* 3A2 exhibited an attenuation profile in mammalian cells and in mice. A single dose of vaccination of *R. parkeri* 3A2 elicited a strong humoral immune response in mice leading to solid protection from lethal challenge with two virulent rickettsial pathogens. *R. parkeri* mutants deserve further evaluation for safety and efficacy to serve as a live-attenuated vaccine against rickettsial diseases in the near future. Our studies on the virulence and immunogenicity of a rickettsial mutant investigated in the mouse model represent novel, promising and valuable data for further evaluating the live-attenuated vaccine candidates against rickettsial diseases and related tick-borne infections.

## 4. Materials and Methods

### 4.1. Construction of Random R. parkeri Transposon Mutants

Plasmid pCis Himar1 A7 was constructed to include mismatched lox5171 and lox2272 sites (designated “lox”) flanking the mCherry (red fluorescent marker) and *aadA* (encoding spectinomycin and streptomycin resistance) genes to facilitate excision of the transposon, pLoxHimar. *Rickettsia parkeri* strain Tate’s Hell was purified from host cells by mechanical disruption with 60/90 rock tumbler grit (Lortone, Mukilteo, WA, USA) and high-speed vortexing. The resulting suspension was passed through a 2-µm pore-size syringe filter, and bacteria were collected by centrifugation at 11,000× *g* for 10 min at 4 °C. Bacteria were washed once in cold 300 mM sucrose, resuspended in 50 µL of the same, and electroporated in the presence of 1 µg of pLoxHimar plasmid DNA (Figure 1A) at 1.8 kV, 400 Ohm and 25 µF using a Gene Pulser II (BioRad, Hercules, CA, USA). Rickettsiae were recovered in 0.3 mL fetal bovine serum (FBS), mixed with ~5 × 10^6 ISE6 tick cells in 1 mL of tick cell culture medium, and centrifuged at 5000× *g* for 5 min at room temperature (RT). The pelleted mixture was incubated for 1 h at 30 °C, and then diluted into 48-well plates containing 5 × 10^4 host cells/well in HEPES/NaHCO_3_-buffered L15C300 medium and incubated at 34 °C in humidified air with 4% CO_2_. Spectinomycin and streptomycin were added to a concentration of 100 μg/mL within 24 h [32]. Subsequently, the culture was fed every two weeks with selection medium and examined weekly for mCherry-expressing rickettsiae using a Diaphot microscope (Nikon, Melville, NY, USA) equipped for epifluorescence. The first fluorescent bacteria containing cells were noted ~3 weeks following electroporation, when the contents of positive wells were transferred to fresh ISE6 cultures in 25 cm^2^ flasks and maintained in selection medium with weekly medium changes until ~90% of cells were infected. At that time, mutants were stored in liquid nitrogen after purification by limiting dilution. To determine insertion sites, the genomic DNA of *R. parkeri* mutants, such as RPATATE_0245::pLoxHimar (3A2) strain, was digested by restriction enzymes HindIII, EcoRI and BglII. These digested gene fragments were ligated into pGEM with compatible ends. Insertion sites were determined by selecting spectinomycin/streptomycin-resistant colonies and sequencing them with primers Ch Up & Out and uv-SS down & out, which read out from either end of the transposon into the interrupted gene. Sequencing was performed at the University of Minnesota Genomics Center.

### 4.2. Genetic Sequencing of R. parkeri 3A2 and Verification of Insertion Region

The insertion of *R. parkeri* mutant 3A2was mapped to the open reading frame of a gene coding for a phage integrase family protein. To confirm the insertion region, specific PCR primers were designed: 3A2 forward (GGCTTGCAAAACGTAACTGTG) and 3A2 reverse (GAGAGCGTAAATCTGCACCA). This primer pair was designed to amplify a 2506 bp fragment of *R. parkeri* 3A2 DNA, which yielded a 612 bp fragment of WT *R. parkeri*. *R. parkeri* 3A2 was grown in Vero cells and DNA was extracted using the DNeasy Blood and Tissue Kit (Qiagen, Hercules, CA, USA). PCR was performed with the following thermocycling parameters: 95 °C for 2 minutes, followed by 35 cycles of 95 °C for 30 s, 53 °C for 30 s, and 72 °C for 3 min. PCR products were subjected to 1.2% agarose gel electrophoresis for visualization.

### 4.3. In Vitro Growth Kinetics of R. parkeri 3A2

We first optimized the concentration of spectinomycin for inhibiting the growth of *R. parkeri* WT but not affecting the viability of host cells. Vero cells in 24-well plate were treated with 10, 50, or 100 µg/mL of spectinomycin. The viability of the cells were determined by the trypan blue exclusion assay. The growth of WT *R. parkeri* in the presence of antibiotics was determined by quantitative real-time PCR as described below. The optimal concentration of spectinomycin to effectively inhibit *R. parkeri* WT growth but not affect the viability of Vero cell was determined to be 100 µg/mL.

To characterize the growth of *Rickettsia*, Vero cells were seeded into a 24-well plate, and then infected with purified *R. parkeri* WT or 3A2 at an MOI of two in a 200 μL of medium with 1% FBS in the absence or presence of spectinomycin. After incubation at RT for 2 h, an additional 800 μL of respective media was added, and plates were continuously incubated at 37 °C with 5% CO_2_ in humidified air for days indicated. Uninfected cells and infected cells in medium without antibiotics served as controls. At the indicated time points, the extracellular rickettsiae were removed by washing with PBS, and the concentrations of rickettsiae were determined via quantitative real-time PCR as described below.

### 4.4. Imaging Fluorescence-Expressing R. parkeri

*R. parkeri* 3A2 was cultivated in Vero cells with medium containing 100 µg/mL spectinomycin. The infection was monitored by Diff-Quik staining. At a proportion of infected cells greater than 75%, a small patch of infected cells was detached using a cell scraper and suspended in 500 µL of SPG buffer solution (218 mM sucrose, 3.76 mM KH2PO4, 7.2 mM K2HPO4, and 4.9 mM glutamate). After obtaining a single cell suspension, cells were centrifuged at 800× *g* for 10 min. Cells were then resuspended in SPG. A drop of live cell suspension was placed onto a slide, covered with a coverslip, and sealed with nail polish before it was examined for red fluorescent intracellular rickettsiae via an Echo Revolve Microscope equipped for epifluorescence and a Texas Red filter (Echo Inc., San Diego, CA, USA) at 60× magnification. Images were acquired with an Apple iPad digital camera and Echo Revolve software (Echo Inc., San Diego, CA, USA).

### 4.5. Culture and Purification of R. parkeri and Its Mutant 3A2 As Well As R. conorii

*R. parkeri* WT, *R. parkeri* mutant 3A2, and *R. conorii* were cultured in Vero cells as previously described [45]. In brief, Vero cells at approximately 100% confluence were infected with *R. parkeri* WT, mutant 3A2, or *R. conorii*, and then cultured in high glucose Dulbecco’s modified Eagle’s medium (DMEM, Gibco Life Technologies, New York, NY, USA) supplemented with 1% heat-inactivated FBS and 1% HEPES buffer (Cellgro, Manassas, VA, USA) in a 37 °C humidified incubator with 5% CO_2_. *R. parkeri* 3A2 was maintained in medium containing 100 µg/mL spectinomycin. Before expansion, *R. parkeri* 3A2 were further purified by plaque assay as described below. Rickettsiae were harvested and purified using a cushion of OptiPrep as described previously [46]. In brief, rickettsiae were sonicated and placed on a 20% OptiPrep density gradient and centrifuged at high speed as described previously [47,48]. The pellet consisting of the purified rickettsiae was resuspended in SPG and stored as stock at −80 °C.

### 4.6. Plaque Assay

Vero cells were seeded in a 24-well plate. A series of 10-fold dilutions of rickettsiae, in 200 μL of DMEM containing 10% FBS, were added to each well containing a confluent monolayer of Vero cells. After cells were incubated with rickettsiae at RT for 6 h, they were placed at 37 °C with 5% CO_2_ overnight before a semi-solid agarose gel overlay was applied on top of the infected Vero cells. After the gel was solidified at RT, the plate was incubated at 37 °C with 5% CO_2_. Plates were monitored daily by the Revolve microscope (Echo Inc., San Diego, CA, USA). After 3–5 days, plaques were imaged and measured with an Apple iPad digital camera attached to the microscope and further analyzed using Echo Revolve software.

### 4.7. Growth of R. parkeri 3A2 in Human Macrophage-Like Cells

THP-1 cells were purchased from ATCC and cultured as previously described without antibiotics [46,49]. THP-1 cells were grown in RPMI medium with 10% FBS and differentiated in medium containing 100 μg/μL phorbol myristate acetate (PMA) (Sigma-Aldrich, St. Louis, MO, USA), reconstituted in dimethyl sulfoxide (DMSO, Sigma-Aldrich) for 16 h followed by 24 h recovery in fresh medium. Cells were plated in a 6-well plate at a density of 1 × 10^6 cells per well followed by infection with *R. parkeri* WT or 3A2 at an MOI of 5. At 3 h, 48 h and 72 h p.i., cells were collected for quantifying the intracellular growth of rickettsiae after washing with PBS as previously described [48].

### 4.8. Quantification of R. parkeri WT and 3A2 by Real-Time PCR

To quantify rickettsiae in either cultured cells or tissues, genomic DNA was extracted using a Qiagen DNA extraction kit (catalog number 69,506; Valencia, CA, USA) as described previously [48]. Quantitative real-time PCR was performed using an iCycler (Bio-Rad, Hercules, CA, USA) as described previously [48]. In brief, quantitative real-time PCR was performed with primers and TaqMan probes for the *Rickettsia*-specific citrate synthase (CS) gene (*gltA*) as described in our previous studies: *gltA* forward, GAGAGAAAATTATATCCAAATGTTGAT; *gltA* reverse, AGGGTCTTCGTGCATTTCTT; *gltA* probe, CATTGTGCCATCCAGCCTACGGT. The *gltA* probe was labeled with 6-carboxyfluorescein (FAM). Two-step cycle parameters (95 °C and 60 °C) were used. The results were normalized to the amount (in nanograms) of genomic DNA in the same sample and expressed as CS gene copy number per nanogram of genomic DNA.

For quantification of rickettsiae in a stock, a 1:100 dilution of rickettsial stock vial was added to each well of a 6-well plate containing a confluent monolayer of Vero cells. The plate was centrifuged at 800× *g* for 5 min, and then incubated at RT for one hour. Next, cells were washed three times with PBS and then incubated at 37 °C in 10% FBS DMEM another hour. After washing again with PBS, cells were collected and processed for real-time PCR to quantify rickettsiae as described above. The copies of CS gene in rickettsial stock were used for the MOI in *in vitro* infection of mammalian host cells.

### 4.9. Evaluation of the Virulence, Immunogenicity and Protective Efficacy of R. parkeri 3A2 In Vivo

Adult (6- to 8-week-old) C3H/HeN mice were purchased from Charles River Laboratories Inc. (Wilmington, MA, USA). To evaluate the virulence and immunogenicity of *R. parkeri* mutant *in vivo*, C3H/HeN mice were inoculated with *R. parkeri* 3A2 i.v. via the tail vein or i.d. at the doses indicated in the figure legends. Mice inoculated with the same dose of *R. parkeri* WT and SPG served as positive and negative controls, respectively. The inoculum of *R. parkeri* WT and 3A2 in mice was determined by quantitative real-time PCR as described above. The inoculation dose of *R. conorii* in mice was determined by plaque assay. The *in vivo* virulence of *R. parkeri* 3A2 was assessed local skin lesion, if mice were inoculated i.d., body weight loss, duration of persistence of rickettsiae and pathological changes in tissues after inoculation. The immunogenicity of *R. parkeri* 3A2 was evaluated by the production levels of IFN-γ and/or IL-10 in serum and the titer of *R. parkeri*-specific antibody in immunized mice before and/or after challenge with WT *R. parkeri.* The efficacy of *R. parkeri* 3A2 was evaluated by the protection conferred by a single dose immunization to mice challenged with a high dose of *R. parkeri* or *R. conorii*.

Following infection, mice were monitored for morbidity (illness score) daily. Illness score was evaluated based on the absence or presence of ruffled fur, hunched back, lethargy, squinty eyes, and inability to reach food or drink, and weight loss (Table 1). 

### 4.10. Cytokine ELISA

Mouse sera were collected and treated with 0.9% sodium azide to inactivate live rickettsiae. The concentrations of IFN-γ and IL-10 in sera were determined by enzyme-linked immunosorbent assay (ELISA) (eBioscience, Invitrogen, San Diego, CA, USA), and absorbance was measured with a VersaMax microplate reader (Molecular Devices, Sunnyvale, CA, USA). The limits of detection of the ELISA for IFN-γ and IL-10 measurement were 5.3 and 32 pg/mL, respectively.

### 4.11. Assessment of Rickettsiae-Specific Antibody Response by IFA

To determine the rickettsiae-specific antibody response in mice immunized with *R. parkeri* 3A2, IFA was performed as described previously [50]. Briefly, *R. parkeri* WT were cultured in Vero cells and deposited on IFA slides. The serum of mice immunized with *R. parkeri* 3A2 were prepared in a 2-fold dilution and overlaid on the slides. After incubation in a humidity chamber at 37 °C for 30 min, slides were washed three times and then dried. Bound antibody was detected with fluorescein-conjugated goat anti-mouse IgG (Vector Laboratories, Burlingame, CA, USA) diluted 1:500 in PBS containing 3% nonfat dry milk and 0.2% Evans blue (Millipore Sigma, St. Louis, MO, USA). The slides were washed and mounted with ProLong™ Gold Antifade Mountant with diamidino-2-phenylindole (DAPI, Invitrogen, Thermo Scientific, Waltham, MA, USA), and examined under an Echo Revolve Microscope equipped for epifluorescence at ×40 magnification.

### 4.12. Evaluation of the Isotype of R. parkeri-Specific IgG Antibody by ELISA

The isotype of WT *R. parkeri*-specific IgG antibody, elicited by immunization with *R. parkeri* 3A2, was determined by an indirect ELISA as described previously with slight modification [51]. Briefly, the microplate was coated with 5 µg/mL of fresh *R. parkeri* WT whole cell lysates, which was inactivated in 0.2% formalin at 4 °C overnight. After washing with 0.05% Tween-20 in 1 × Dulbecco’s PBS (DPBS), the plate was blocked with DPBS containing 0.1% Tween-20, 1% bovine serum albumin (BSA). After 2 h, sera were diluted, and then added to the plates followed by incubation at RT for 2 h. Diluted goat anti-mouse IgG1 or IgG2a antibody (1:5000) (Southern Biotech, Birmingham, AL, USA) was added and incubated for another 2 h. After washing, tetramethylbenzidine (TMB) substrate solution (Invitrogen) was added to the wells and the reaction was terminated by stop solution (2N H_2_SO_4_). Plates were immediately read at 450 and 570 nm using a VersaMax microplate reader (Molecular Devices). The results were reported as the reciprocal of the highest titer giving an optical density (O.D.) reading of at least mean +2SD greater than the sera from mice inoculated with PBS. All assays were performed in triplicate, and results were shown as the mean reciprocal endpoint titer.

### 4.13. Histopathological Analyses

Formalin-fixed, hematoxylin and eosin (H&E)-stained tissue sections from infected and uninfected mice were evaluated by a pathologist at ×10, ×20, ×40 and ×60 optic magnification using an Echo Revolve Microscope (Echo Inc.) equipped for bright field microscopy. Images were taken with an Apple iPad digital camera attached to the Echo Revolve Microscope. Images were analyzed for both frequency and size of inflammatory infiltration foci using Echo Revolve software.

### 4.14. Statistical Analysis

One-way analysis of variance (ANOVA) with Bonferroni’s procedure was used for comparison of multiple experimental groups and analyzed statistically with GraphPad Prism software version 9.1.1 (GraphPad Software, San Diego, CA, USA). T-test was used to compare the outcomes between two groups at the specific time point(s). *p* values of 0.05 or less were considered significant. Survival differences were compared using Kaplan-Meier survival curves, followed by a log rank test. For comparison of the growth kinetics of *R. parkeri* WT and 3A2 (Figure 2A,C), due to the violation of normality assumption for ANOVA, we analyzed these results using non-parametric ANOVA–Friedman test, and Mann-Whitney test. Two-way ANOVA and Friedman test including main effects of group and days, and the interaction between group and day was used to compare the difference in the growth of outcome between two experimental groups.

## Figures and Tables

**Table 1:** Mouse illness score.

Score Illness	0	1	2	3	4	5
Ruffled Fur	A	A	P	P	P	P
Hunched Back	A	A	A	P	P	P
Lethargy	A	A	A	P	P	P
Squinty Eyes	A	A	A	A	P	P
Inability to Reach Food/Drink	A	A	A	A	P	P
Weight Loss	A	<5%	6–10%	11–15%	16–20%	>20%

Mice were assessed every day and scored for individual symptoms, including ruffled fur, hunched back, lethargy, squinty eye, inability to reach food or drink, and weight loss. A, absent; P, present. The minimum score was 0 for a healthy mouse and 1~5 for sickness, depending on severity. Mice with scores of 4~5 were considered moribund and were humanely euthanized.

## Data Availability

Not applicable.

## Supplementary Materials

The following are available online at https://www.mdpi.com/article/10.3390/pathogens10070819/s1, Figure S1: Dose-dependent mouse model of *R. parkeri* rickettsioses, Figure S2: IFA of *R. parkeri* WT antigen with sera from mouse immunized with *R. parkeri* 3A2.

## Abbreviations

*R. parkeri* 3A2, *Rickettsia parkeri* mutant RPATATE_0245::pLoxHimar; CS, citrate synthase; WT, wild type; ns, not statistically significant; i.v., intravenous; i.d., intradermal; p.i., post infection; SPG, sucrose-phosphate-glutamate; PBS, phosphate-buffered saline; ELISA, enzyme-linked immunosorbent assay; p.c., post challenge; D1, day 1; D4, day 4; FBS, fetal bovine serum; RT, room temperature; PMA, phorbol myristate acetate; DMSO, dimethyl sulfoxide; h, hour; DMEM, Dulbecco’s modified Eagle’s medium; IFA, indirect immunofluorescence assay; DAPI, diamidino-2-phenylindole; BSA, bovine serum albumin; O.D., optical density; ANOVA, one-way analysis of variance; DPBS, dulbecco’s phosphate buffered saline.
