# Supplementary material for: Rickettsia parkeri with a Genetically Disrupted Phage Integrase Gene Exhibits Attenuated Virulence and Induces Protective Immunity against Fatal Rickettsioses in Mice
Source: Pathogens. 2021 Jun 30;10(7):819. doi: 10.3390/pathogens10070819 (PMC8308654; doi:10.3390/pathogens10070819)
Supplement: Supplementary file 1 [file pathogens-10-00819-s001.zip › pathogens-1195054-supplementary.pptx]

## Slide 1
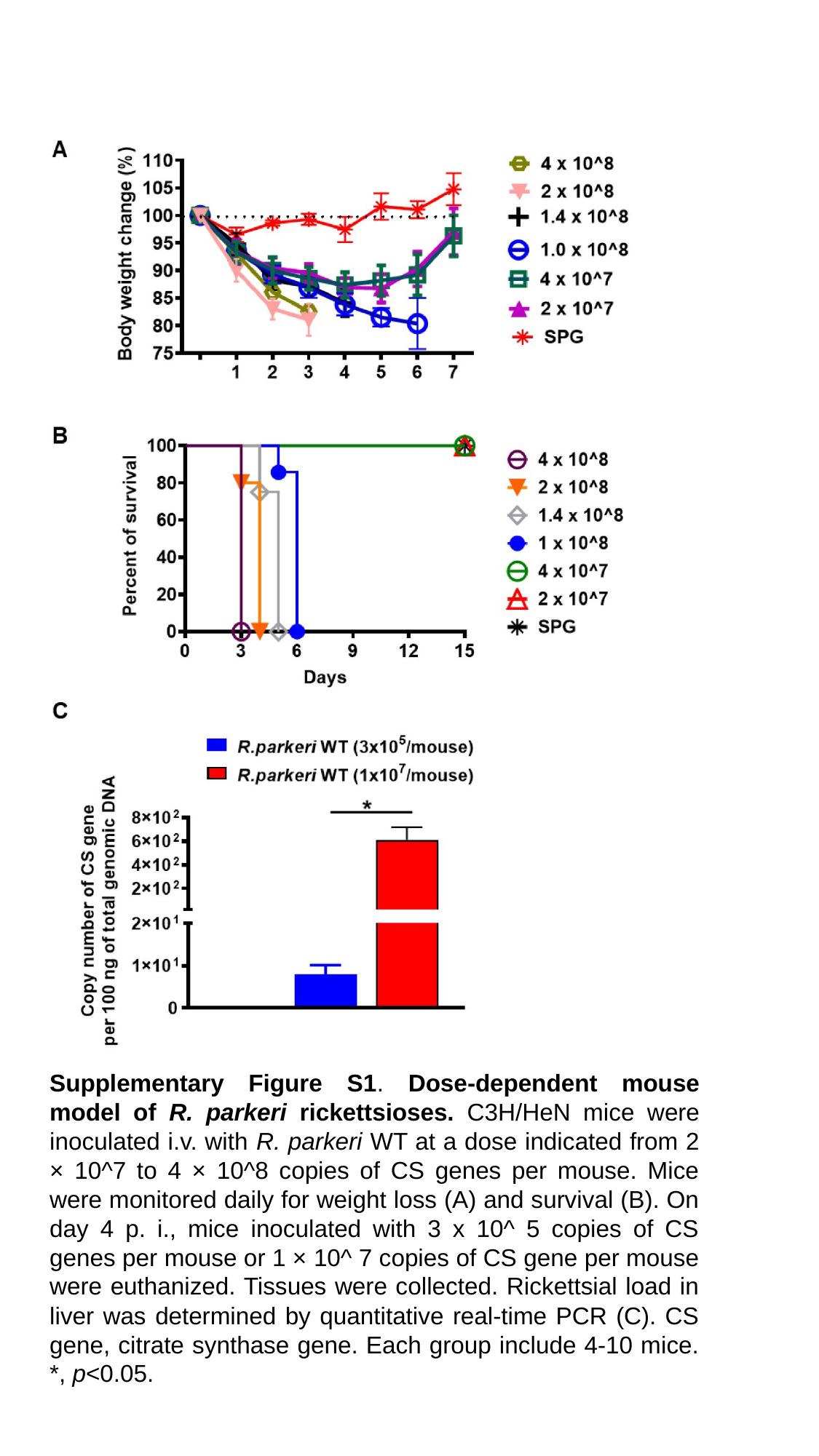

Supplementary Figure S1. Dose-dependent mouse model of R. parkeri rickettsioses. C3H/HeN mice were inoculated i.v. with R. parkeri WT at a dose indicated from 2 × 10^7 to 4 × 10^8 copies of CS genes per mouse. Mice were monitored daily for weight loss (A) and survival (B). On day 4 p. i., mice inoculated with 3 x 10^ 5 copies of CS genes per mouse or 1 × 10^ 7 copies of CS gene per mouse were euthanized. Tissues were collected. Rickettsial load in liver was determined by quantitative real-time PCR (C). CS gene, citrate synthase gene. Each group include 4-10 mice. *, p<0.05.

## Slide 2
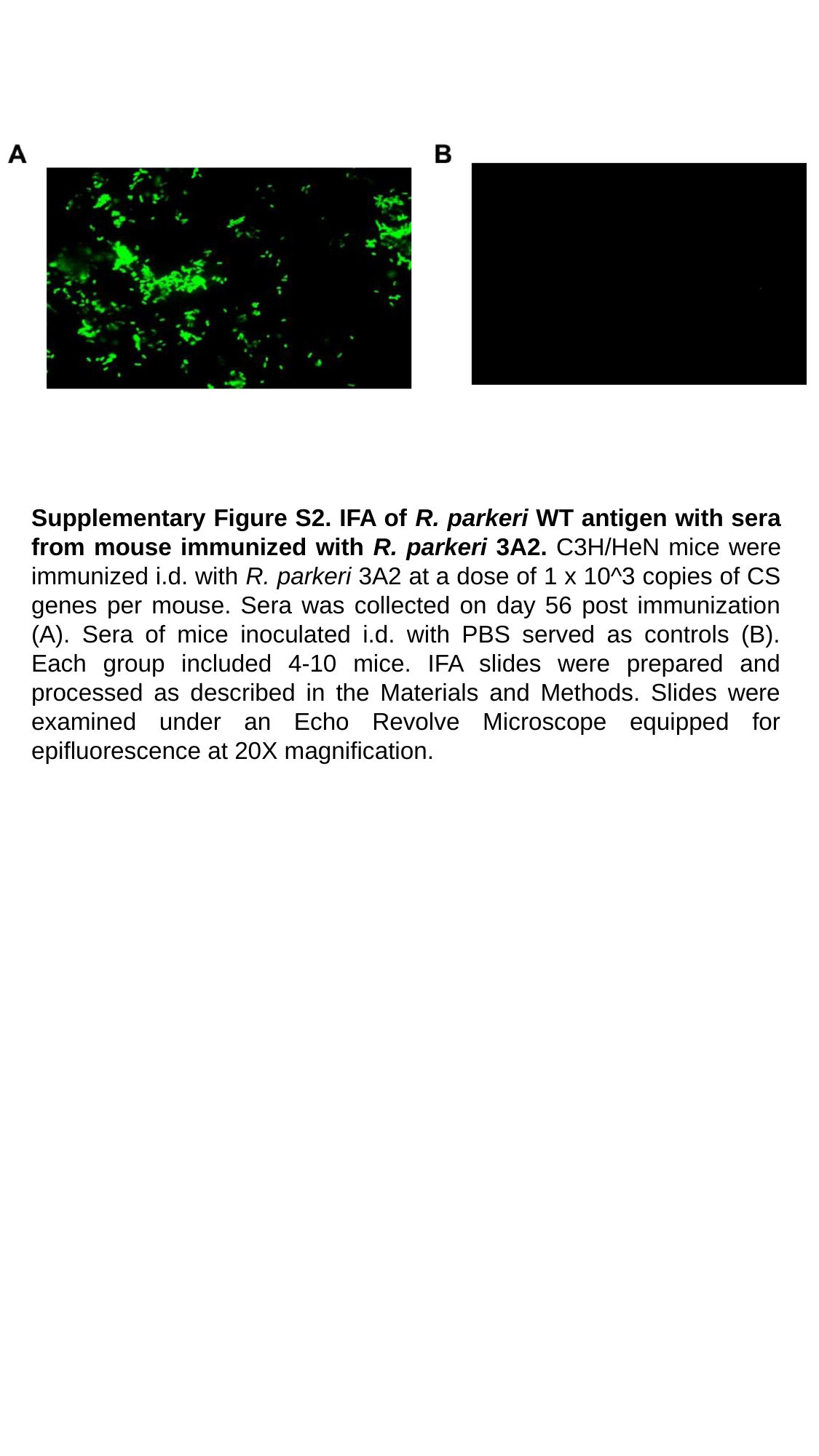

Supplementary Figure S2. IFA of R. parkeri WT antigen with sera from mouse immunized with R. parkeri 3A2. C3H/HeN mice were immunized i.d. with R. parkeri 3A2 at a dose of 1 x 10^3 copies of CS genes per mouse. Sera was collected on day 56 post immunization (A). Sera of mice inoculated i.d. with PBS served as controls (B). Each group included 4-10 mice. IFA slides were prepared and processed as described in the Materials and Methods. Slides were examined under an Echo Revolve Microscope equipped for epifluorescence at 20X magnification.
